# An interferometric ex vivo study of corneal biomechanics under physiologically representative loading, highlighting the role of the limbus in pressure compensation

**DOI:** 10.1186/s40662-020-00207-1

**Published:** 2020-08-13

**Authors:** Abby Wilson, John Jones, John R. Tyrer, John Marshall

**Affiliations:** 1 Wolfson School of Mechanical, Manufacturing and Electrical Engineering, Loughborough University, Loughborough, UK; 2 Laser Optical Engineering Ltd., Derbyshire, UK; 3 Institute of Ophthalmology, UCL, London, UK

**Keywords:** Cornea, Biomechanics, Interferometry, Limbus, Topography

## Abstract

**Background:**

The mechanical properties of the cornea are complex and regionally variable. This paper uses an original method to investigate the biomechanics of the cornea in response to hydrostatic loading over the typical physiological range of intra-ocular pressure (IOP) fluctuations thereby increasing understanding of clinically relevant corneal biomechanical properties and their contributions to the refractive properties of the cornea.

**Methods:**

Displacement speckle pattern interferometry (DSPI) was used to measure the total surface displacement of 40 porcine and 6 human corneal-scleral specimens in response to pressure variations up to 1 mmHg from a baseline of 16.5 mmHg. All specimens were mounted in a modified artificial anterior chamber (AAC) and loaded hydrostatically. Areas of high strain in response to loading were identified by comparing the displacements across different regions.

**Results:**

The nature of the response of the corneal surface to loading demonstrated high regional topographic variation. Mechanical properties were shown to be asymmetrical, and deformation of the limbal and pre-limbal regions dominated these responses respectively with over 90% (N-T) and 60% (S-I) of the total maximum displacement occurring in these regions indicating high-strain. In contrast, the curvature of the central cornea remained relatively unchanged merely translating in position.

**Conclusions:**

The limbal and pre-limbal regions of the cornea appear to be fundamental to the absorption of small pressure fluctuations facilitating the curvature of the central cornea to remain relatively unchanged. The differential mechanical properties of this region could have important implications for the application of corneal surgery and corneal crosslinking, warranting further investigation.

## Introduction

The biomechanics of the cornea govern its shape and hence its refractive power. Understanding how the mechanical properties of the cornea contribute to its shape has become of increasing importance since the advent and rapid dissemination of refractive surgery in the 1990’s, as the introduction of surgical incisions to the cornea results in modifications to its mechanical properties [1, 2]. It is now even more pertinent given the possibility of direct topographic manipulation of corneal stiffness via corneal crosslinking (CXL) [3], which has demonstrated potential as a minimally-invasive means to provide small refractive correction [4]. However, there have been major limitations associated with the techniques employed for biomechanical assessment in basic and clinical studies, and to date, there is no established method for evaluating the mechanics of the cornea and generating spatially resolved information that is relevant to the clinician.

The cornea is a structurally complex, viscoelastic membrane with regionally variable, strain dependent biomechanical properties; hence, many factors such as the strain rate, hydration and direction of the applied force, can lead to variations in response [5]. To gain a useful understanding of how biomechanics govern corneal shape and behaviour and predict how the biomechanical properties of the tissue may change following disease, trauma or surgical intervention, it is necessary to consider the mechanics of the entire cornea in response to physiologically-relevant forces.

However, up to the present time, the majority of studies on corneal biomechanics have been performed ex vivo*,* using strip extensometry to evaluate corneal elasticity expressed in terms of the elastic moduli (Young’s, tangent, secant) of excised strips of corneal tissue [6–8]. Whilst this technique has advantages in terms of its simplicity, low cost, and ability to provide a quantitative measure of stiffness, it also has many limitations. Principally, strips are isolated from the dome of the cornea, compromising the integrity of its microstructure, and removing the natural physiological state of tension and shape that plays an important role in governing the biomechanics of the tissue in vivo. This, in addition to the difficulties associated with maintaining a strip of tissue at physiological hydration during the measuring procedure; and the fact strains generally far exceed those experienced physiologically contributes to the substantial variation in elastic moduli that have been reported in the literature, with values ranging from 1.3 MPa [9] to 57 MPa [10]. Hence, despite extensometry testing providing a useful and simplistic measure of changes to tissue elasticity in the case of interventions such as CXL, it would be erroneous to assume that stiffness and tensile properties measured via this method are truly applicable to whole-tissue models of corneal biomechanics.

Acknowledging the limitations of strip testing, a variety of methods have now been investigated to probe the biomechanics of intact corneal tissue under physiologically relevant loading conditions, these methods include; Brillouin spectroscopy (BrS), optical coherence elastography (OCE), high frequency ultrasound (HFU), digital image correlation (DIC) and interferometry. Of these, BrS and OCE have demonstrated potential to acquire data in a clinical setting [11, 12]. BrS evaluates longitudinal modulus, which is related to compressibility (bulk modulus) in isotropic materials. However, in hydrated materials such as the cornea, BrS signals represent a volume-weighted aggregate longitudinal modulus of the fluid and solid components of a tissue, often dominated by the fluid component (which for the cornea is spatially and temporally variable). As a result, Brillouin spectra do not correlate directly with elastic properties, recently leading to one group cautioning against its use as an optical elastography tool for biological materials [13].

Furthermore, both BrS and OCE are scanning based methods, so there is an inherent trade-off between spatial resolution and acquisition time, with BrS requiring several minutes to generate a relatively low-resolution, limited area, 40-point scan [11]; and OCE requiring around 5 min to obtain high-resolution 3D data [14]. A more prominent limitation of OCE is a loss of speckle contrast at the corneal periphery limiting data acquisition to the central ~ 8 mm [14], unless the cornea is rotated with respect to the image system, leading to longer acquisition times and complexities with image stitching during post-processing. HFU has recently demonstrated potential to measure corneal strain in response to changes in IOP over the cardiac cycle [15], however this method also requires scanning, with data only presented so far for the central region of 2D cross-sections [15, 16]. If clinical translation is to be realised using either OCE or HFU, further work is required to extend the region of measurement and to account for eye movement over the measurement time.

In contrast to the above methods, DIC and Interferometry are snapshot methods where whole-field data can be captured over a single measurement, requiring only milliseconds. DIC has successfully been used ex vivo to examine regional differences in surface displacement in response to loading [17, 18], however displacement sensitivity is limited relative to interferometric techniques. Using speckle interferometric methods, it is possible to generate high spatial resolution maps of corneal and scleral surface deformation, with a displacement sensitivity of 10’s of nanometres, over a single measurement taking only milliseconds. Displacement speckle pattern interferometry (DSPI) has previously been used for the examination of ovine corneas [1], where it was used to quantify the effects of refractive surgery on biomechanics. Radial speckle pattern shearing interferometry has also been employed to examine age related stiffening [19] and the biomechanical effects of CXL [3], however this particular method is inherently limited by a lack of sensitivity at the central cornea. Despite significant advantages in terms of acquisition time, both DIC and interferometry measure the surface movement, hence the bulk movement of the cornea; they cannot provide detail on any compression that may occur through the thickness of the tissue.

Clinical assessment of corneal biomechanics currently relies on evaluation of the response of the cornea to an air-puff directed at its centre, either via the ocular response analyser (ORA) [20] or the dynamic scheimpflug tonometer (DST) [21]. Although DST has recently demonstrated efficacy for the early detection of biomechanical abnormality [22], neither of these methods directly asses corneal stiffness and cannot realistically predict movement parameters outside the location of the air-puff.

In the present study, DSPI was used to obtain non-contact, non-destructive measurement of full-corneal surface deformation in response to hydrostatic pressure changes designed to simulate small changes in IOP, such as those that occur during the cardiac cycle [23]. Building upon previous work [1], where only a single component of deformation was analysed, here DSPI was combined with subtractive cross-sectional imaging enabling the total set of deformation to be estimated.

## Methods and materials

### Measurement and loading system

The working principles of DSPI are diagrammatically summarised in Supplementary Figure 1. During DSPI, the surface of the target is illuminated with coherent light. In the presence of surface height variations greater than the wavelength of the illumination source, light scattered from the surface of interest has a speckled appearance. The light scattered from this surface is interfered with that of a reference and imaged on a detector to create an interferogram. The speckle pattern formed is unique to that object at the time of measurement, should the surface move, this speckle pattern will change. Capturing a speckle pattern of the object in its reference state and then digitally subtracting subsequent speckle patterns captured as the object moves results in the formation of interference patterns consisting of light and dark fringes, which, with knowledge of the optical set-up, can be mathematically analysed to quantify the surface displacement [24], which when combined with phase-shifting provides sensitivity to displacement on a scale of 10’s of nanometres [25].

**Fig. 1:**
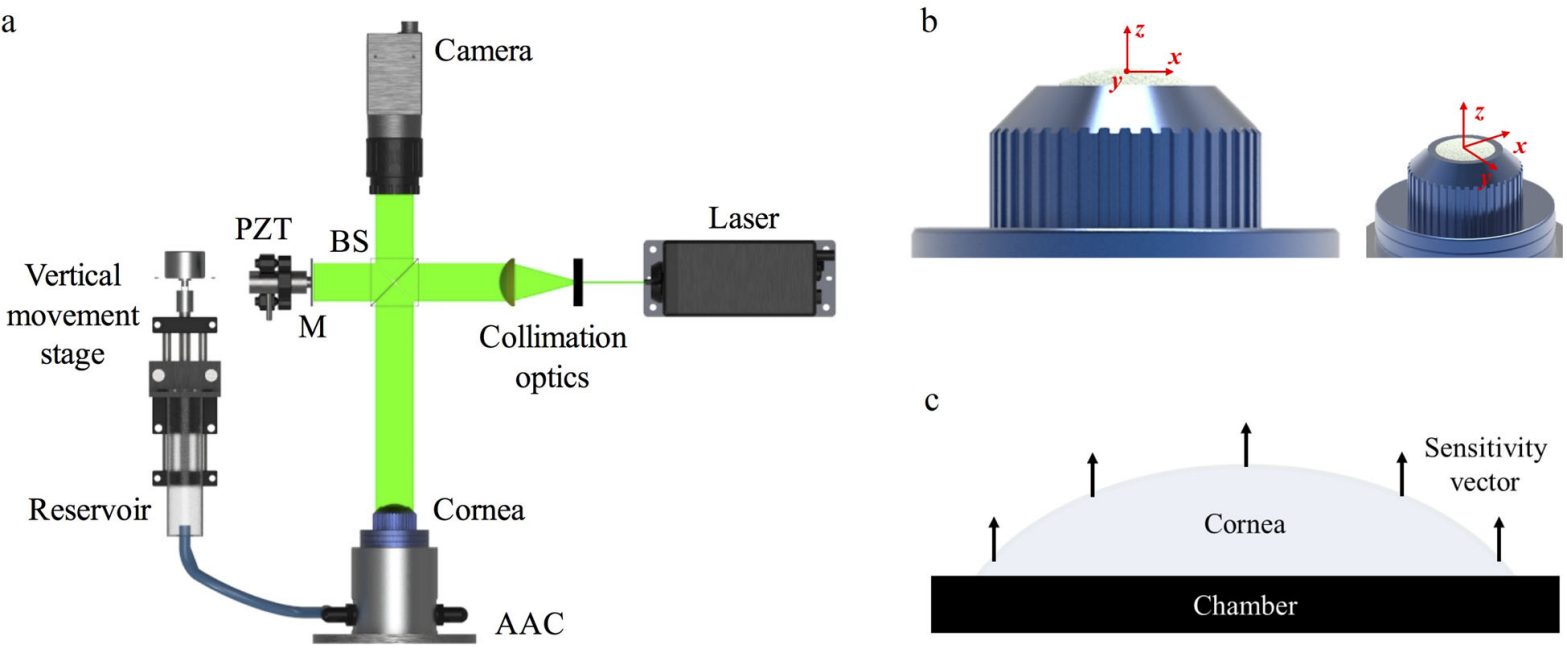
Schematics showing the layout of the DSPI system (BS – Beamsplitter, PZT – Piezoelectric Transducer, M – Mirror) (**a**), the AAC onto which corneas were mounted during testing (**b**), and the direction of measured displacement across the corneal surface (**c**). Out-of-plane displacement (w) corresponds to displacement along the direction of the z-axis, horizontal in-plane (u) and vertical in-plane (v) correspond to displacement along the direction of the x and y axes, respectively

The layout of the interferometer that was used in this study for the measurement of surface deformation, and the artificial anterior chamber (AAC) onto which corneas were mounted are shown in Fig. 1a and b, respectively. For the interferometry system, illumination was via a diode pumped single-mode solid-state laser (*λ* = 532 nm) (06-DPL, Cobolt AB, Solna, SE). The laser was expanded and collimated to a diameter of 25 mm. The illumination beam was passed through a 50:50 beamsplitter with half directed towards the corneal surface and half towards a planar mirror attached to a piezo-electric transducer which was used to generate a phase-stepped reference beam. The beams from the object and the reference were imaged using a CMOS camera with a resolution of 1296 by 972 pixels (CMOS Aptina MT9P031, Basler AG, Ahrensburg, DE) through a 12.5 mm – 75 mm zoom lens (C31204, Pentax, Tokyo, JP).

The configuration of the interferometric system, with illumination and imaging directed normal to the surface of the target dictates that the sensitivity is to out-of-plane deformation only (Fig. 1c) and this is constant across the surface of the object. However, due to the curved nature of the cornea, the vector of total displacement is likely to vary across the surface. Therefore, to completely define the deformation of the corneal surface, it was necessary to define the out-of-plane and horizontal and vertical in-plane components of deformation. This was impractical to achieve using interferometry on a curved surface, due to the requirement for off-axis illumination and imaging, hence, problems with shading and uneven illumination. Consequently, estimations for the in-plane component of deformation were made using subtractive imaging of the central cross-section of a cornea deforming under a larger change of pressure (< 20 mmHg) and tracking the angle of surface movement via tracking the movement of recognisable regions on the corneal surface, identifiable due to the roughness of the applied surface coating.

Pressure in the AAC was controlled via a reservoir supplying fluid to the closed chamber. During testing, the reservoir and the chamber were filled with Phosphate Buffered Saline (PBS) solution (Sigma-Aldrich, UK, *ρ* = 0.995 g/ml at 25 °C). The reservoir was raised 225 mm above the surface of the cornea to provide a baseline pressure of 16.5 mmHg, within the representative range of normal IOP in both human and porcine eyes [26–28]. To raise and lower the pressure from this baseline, the height of the chamber was adjusted electronically via attachment of the reservoir to a motorised vertical translation stage capable of pressure variations up to a maximum of 4 mmHg above the baseline pressure.

### Validation experiments

To establish that the interferometer was functioning correctly, prior to corneal measurement, two initial experiments were conducted. The first experiment was on a circularly clamped steel plate, centrally point-loaded with a micrometre screw. A pre-load was applied, and a reference image was acquired, the plate was then displaced by 1 μm at the centre via movement of the micrometre screw and data was acquired, 10 repeated measurements were taken. Following this, measurements were conducted on a curved silicone hemispherical membrane of uniform thickness (Mini Semisphere mould, Silikomart, IT, r_c_ = 15 mm) mounted in the AAC to check that the AAC did not induce any abnormal boundary effects. In this case the membrane was set under an initial pressure of 16.50 mmHg and hydrostatic pressure was increased by 1 mmHg over the course of measurement.

### Corneal preparation and testing

Fresh porcine eyes (< 12 h post-mortem) with a clear cornea and intact epithelium were obtained from a local abattoir (Joseph Morris Butchers, South Kilworth, UK). The eyes were stored in a moist, sealed container and refrigerated at 4 °C until measurement. All porcine eyes were used within 3 days of slaughter. Prior to measurement, the corneas were isolated from the posterior globe, leaving a 3 mm scleral border for mounting. For the human eye experiments, 6 human corneal-scleral specimens from 3 donors were obtained from Moorfield’s Biobank (UCL Institute of Ophthalmology, London, UK), with ethical approval granted by Moorfields Biobank internal ethics committee (Ref: 10/H0106/57-2015ETR45). The human corneas were surplus for donor purposes and had been collected with permission for research use. The corneal specimens had been in storage for over 8 weeks prior to being released for research use. The human corneas were suspended in organ donor culture (80 ml Eagle’s minimum essential medium with HEPES buffer, 26 mmol/l NaHCO3, 2% foetal bovine serum, 2 mmol/l L-glutamine, penicillin, streptomycin and amphotericin B). All corneas remained in the solution until required for measurement.

All corneas were de-epithelialized prior to measurement. The epithelium has previously been shown to have a negligible contribution to stiffness [29], and its removal prevented any changes to the epithelium, that may have occurred during storage, from affecting the measured response. The corneal-scleral specimens were then mounted within the AAC, which imposed a fixed boundary on the scleral region 1.5 mm to 3.0 mm outside the edge of the cornea. As the cornea is designed to transmit optical radiation, surface reflection was small and had to be enhanced by means of a scattering agent. The corneas were coated with a thin layer of hollow glass microspheres - Sphericel 110P8 (Potters Ind. LLC, PA, USA), to amplify the scatter from the surface. Prior to loading and measurement, the corneas were set under the baseline pressure of 16.5 mmHg and maintained under this constant pressure for 30-min to allow for stress-relaxation. During testing, each cornea underwent 3 loading cycles during which the pressure was raised by 0.25, 0.5, 0.75 and 1 mmHg in turn. At the end of each loading increment, the movement of the reservoir was paused for 0.5 s during which data was captured and processed, and after which the pressure was restored to baseline.

### Data analysis

The out-of-plane displacement (*w*) was calculated from the measured phase change due to deformation (*∆ϕ*_*def*_ (radians)) using eq. 1 [24], where the wavelength of the illumination source (*λ*) was 532 nm.

1 $$ w=\Delta  {\phi}_{def}.\frac{\lambda }{4\pi } $$

The horizontal in-plane (u) and vertical in-plane (v) displacement components were estimated using the measured out-of-plane displacement component (w) and the angle of deformation estimated from subtractive cross-sectional imaging as described by eqs. 2 and 3, where θ_x_ and θ_y_ are the angles of deformation with respect to the horizontal and vertical axes, respectively.

2 $$ u=w\ \tan {\theta}_x $$

3 $$ v=w\ \tan {\theta}_y $$

## Results

### Loading plate and response of uniform membrane

**Fig. 2:**
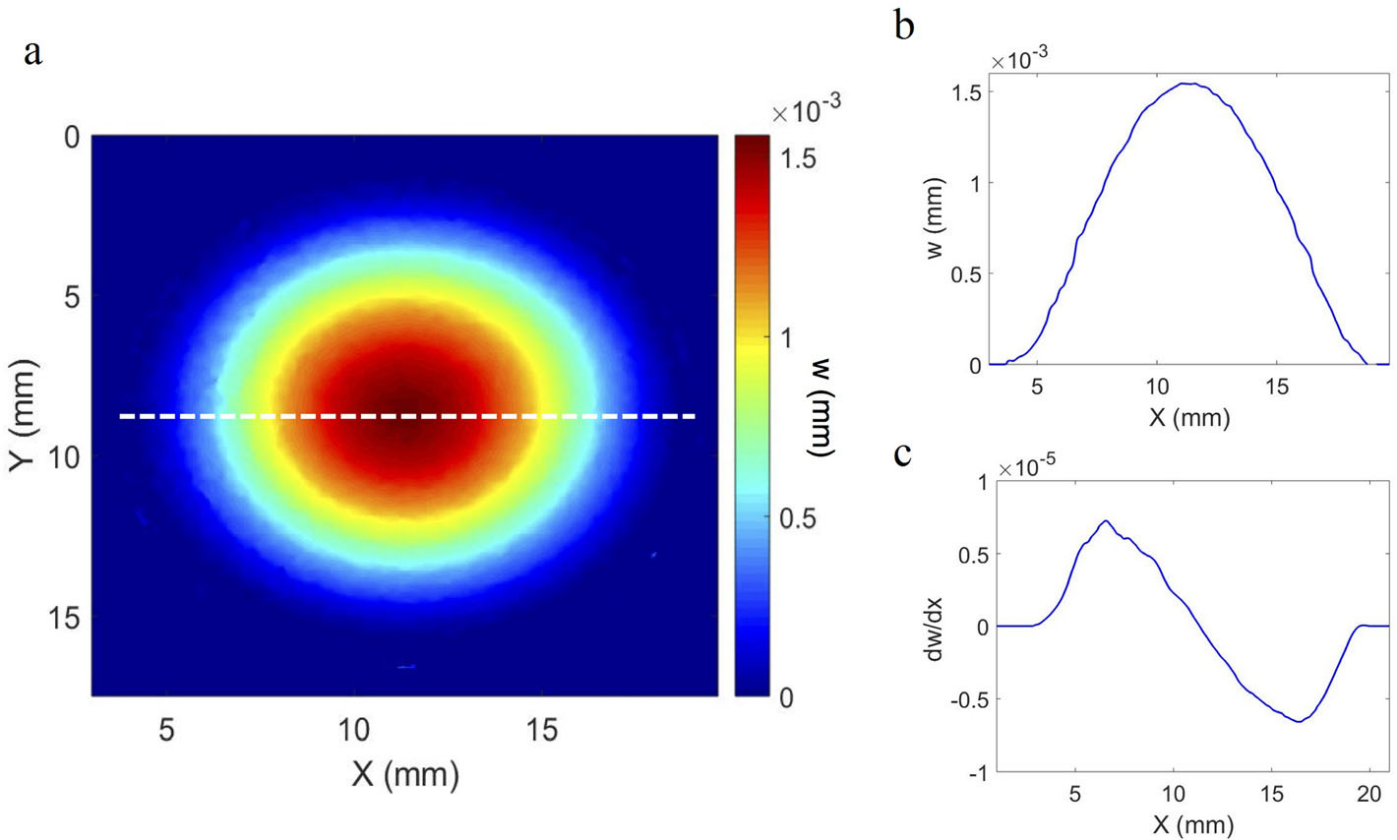
Out-of-plane surface displacement of a curved silicone hemispherical membrane in response to hydrostatic pressure increase from 16.5 mmHg to 17.5 mmHg; whole surface displacement map (**a**); cross-section of out-of-plane displacement along dashed line (**b**); cross-section of gradient of out-of-plane displacement along dashed line (**c**)

The average central displacement of the clamped steel plate loaded via the micrometre screw was as expected at 0.98 μm (SD ± 0.02 μm). The deformation of the curved homogenous membrane (Fig. 2a) also showed the expected distribution, with zero deformation at the fixed boundary, maximum displacement at the central point furthest from the fixed boundary (Fig. 2b) and a relatively constant gradient to the rate-of-change of displacement (Fig. 2c).

### Regional differences in corneal response

**Fig. 3:**
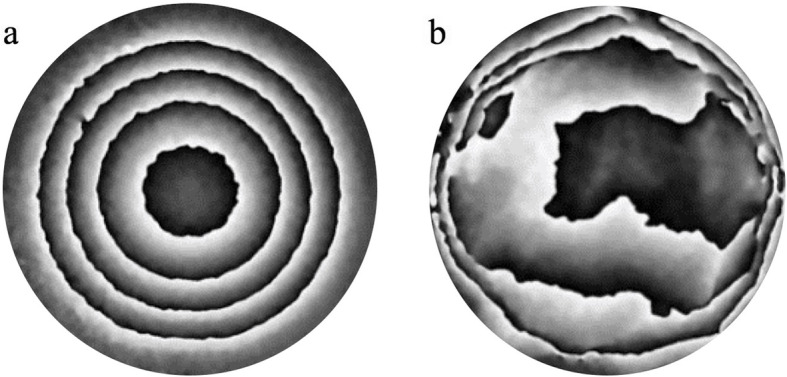
Fringe distributions obtained from a curved silicone hemispherical membrane (**a**) and a human cornea (**b**) in response to a pressure increase from 16.5 mmHg to 17.0 mmHg

**Fig. 4:**
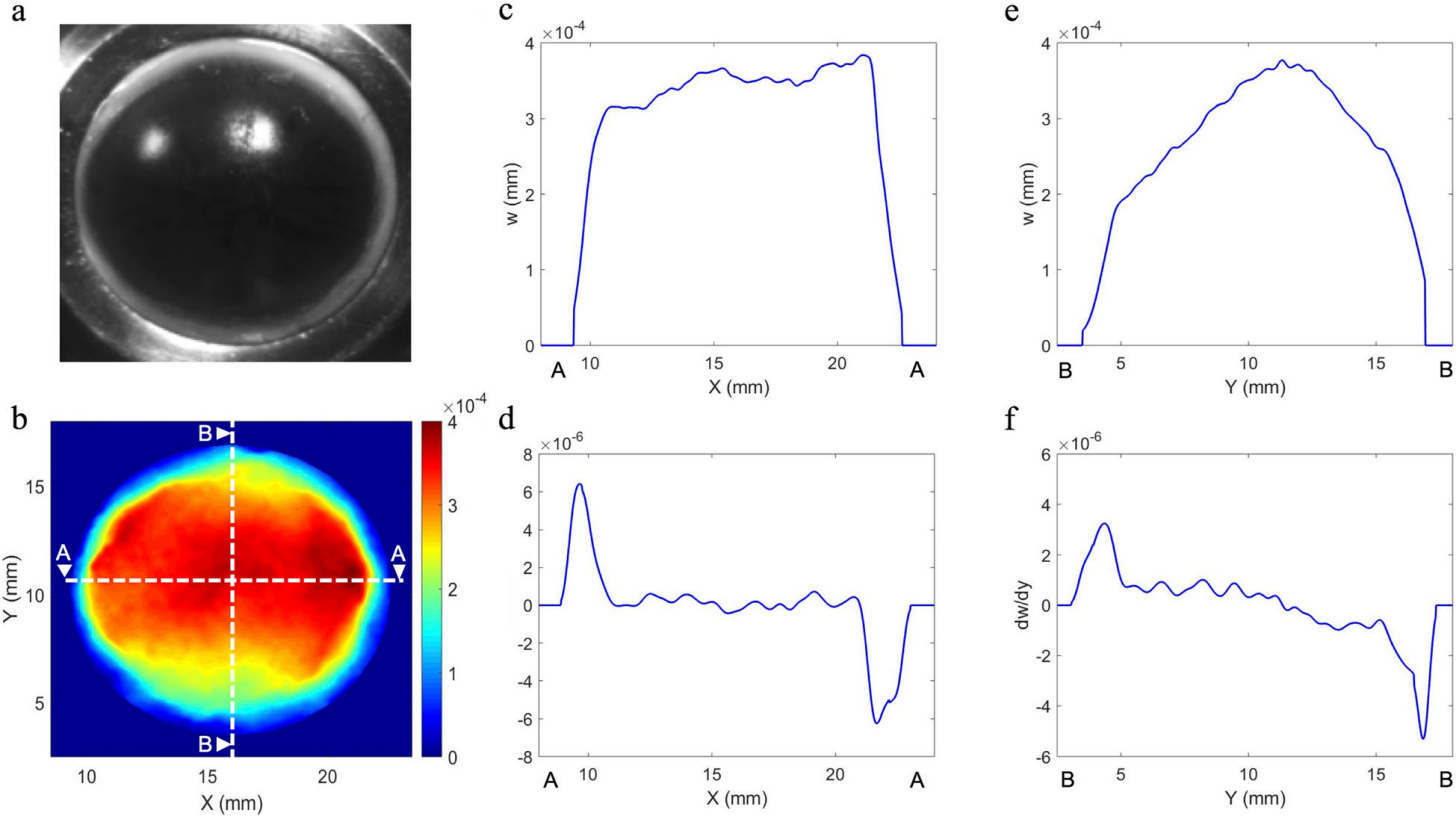
Typical response of a human cornea to a pressure increase from 16.5 mmHg to 16.75 mmHg. Photograph of cornea in AAC for positional reference (**a**), full-surface map of out-of-plane displacement (**b**); out-of-plane displacement across section A-A (**c**); gradient of out-of-plane displacement across section A-A (**d**); out-of-plane displacement across section B-B (**e**); gradient of out-of-plane displacement across section B-B (**f**)

In contrast to the homogenous membrane (Figs. 2 and 3a), corneas were found to demonstrate high regional variability in response to pressure changes (Figs. 3b and 4). In both the human and porcine corneas examined, there was a tendency for deformation to be concentrated in the limbal region. This was evident due to the high fringe concentration observed in this region, relative to other regions during loading (Fig. 3b) which was in clear contrast to the response of the homogenous membrane, which when mounted in the AAC, showed relatively even fringe spacing (Fig. 3a). Furthermore, a common trend was observed with 23/40 (57%) porcine corneas and 4/6 (67%) of human corneas showing similarities in the pattern of regional displacement.

A typical response of a human cornea is shown in Fig. 4 (responses of all human corneas are provided in Supplementary Figures 2 and 4). In these specimens, the response was asymmetrical; the rate of change of displacement across the central 8 to 10 mm of the cornea was close to zero (Table 1), especially across the nasal-temporal axis (Fig. 4d) indicating minimal changes in curvature across this region; and the highest rate of change of displacement was at the edge of the cornea in the limbal region (Fig. 4d and f), where the cornea joins the sclera. Taken together, these indicate a high strain concentration at the limbal/prelimbal region, confirmed by the relatively higher values for $\frac{\delta w}{\delta x}$ and $\frac{\delta w}{\delta y}$ here when compared to the central 8 mm of the N-T and S-I axes (Table 1). It was also observed that while the magnitude of $\frac{\delta w}{\delta x}$ and $\frac{\delta w}{\delta y}$ increased significantly in the limbal region in response to larger pressure variations (0.5 to 1.0 mmHg), across the centre $\frac{\delta w}{\delta x}$ and $\frac{\delta w}{\delta y}$ remained almost constant, showing minimal changes to the curvature of this region across the full pressure range. In general, over 90% (N-T axis) and 60% (S-I) of the maximum out-of-plane displacement occurred in the outer 2 mm of the human corneas incorporating the limbal region (Table 1). A typical response for a porcine cornea is shown in Supplementary Figure 3. As with human corneas, the majority of strain was concentrated at the limbus however, in porcine corneas, deformation was greater in the peripheral cornea at the superior and inferior poles when compared to the nasal temporal.

**Table 1:** Comparison of out-of-plane displacement of human corneas at the central cornea and at the limbus in response to a 0.25 mmHg increase in hydrostatic pressure

Human cornea No.	w at central cornea, mean ± SD (μm)	w at limbal/prelimbal region (N-T axis) (μm)	w at limbal/prelimbal region (S-I axis) (μm)	Mean dw/dx (central 8mm N-T axis) ×1.0e^-06^	Mean dw/dx (limbus N-T axis) ×1.0e^-06^	Mean dw/dy (central 8mm S-I axis) ×1.0e^-06^	Mean dw/dy (limbus S-I axis) ×1.0e^-06^
1	0.46 ± 0.058	0.46	0.44	0.66	3.39	0.74	3.59
2	0.29 ± 0.036	0.27	0.20	0.31	4.21	0.57	2.88
3	0.35 ± 0.028	0.37	0.27	0.26	4.39	0.57	1.91
4	0.68 ± 0.050	0.46	0.42	1.22	3.87	1.16	3.91
5	0.71 ± 0.068	0.66	0.63	0.58	5.70	0.81	5.13

The corneas that did not show this typical distribution of displacement can be grouped into two main categories. A few porcine corneas (5/40) showed a similar displacement distribution to the one previously described, but rotated through 90 °, suggesting that this could have occurred due to errors in determining initial corneal orientation. The majority of the other corneas tested (12/40 porcine corneas and 2/6 human corneas), showed a distribution where the maximum rate of change of displacement remained at the limbal region, but instead of maintaining an overall relatively constant level of displacement across the central region, the displacement tended to be greater in one particular region, indicating localised regions of biomechanical weakness. The position of this region varied between corneas. In one human cornea, this area of weakness was clearly identifiable as damage introduced from the insertion of the surgical thread used to suspend the cornea in the transplant solution (Supplementary Figure 4). Discounting this cornea, the magnitude of out-of-plane displacement at the central cornea for the remaining human corneas tested in response to a 0.25 mmHg change in hydrostatic pressure are shown in Table 1 and ranged from 0.29 μm to 0.71 μm (median 0.50 μm). Assuming linearity in response, this corresponds to apical displacement of 3.6 μm to 10.4 μm over a normal range of ocular pulse amplitudes (1.8–4.3 mmHg) [23] consistent with a recent study examining the deformation of a whole eye-globe over a physiological range of pressure variations [18].

### Directional aspects of response

**Fig. 5:**
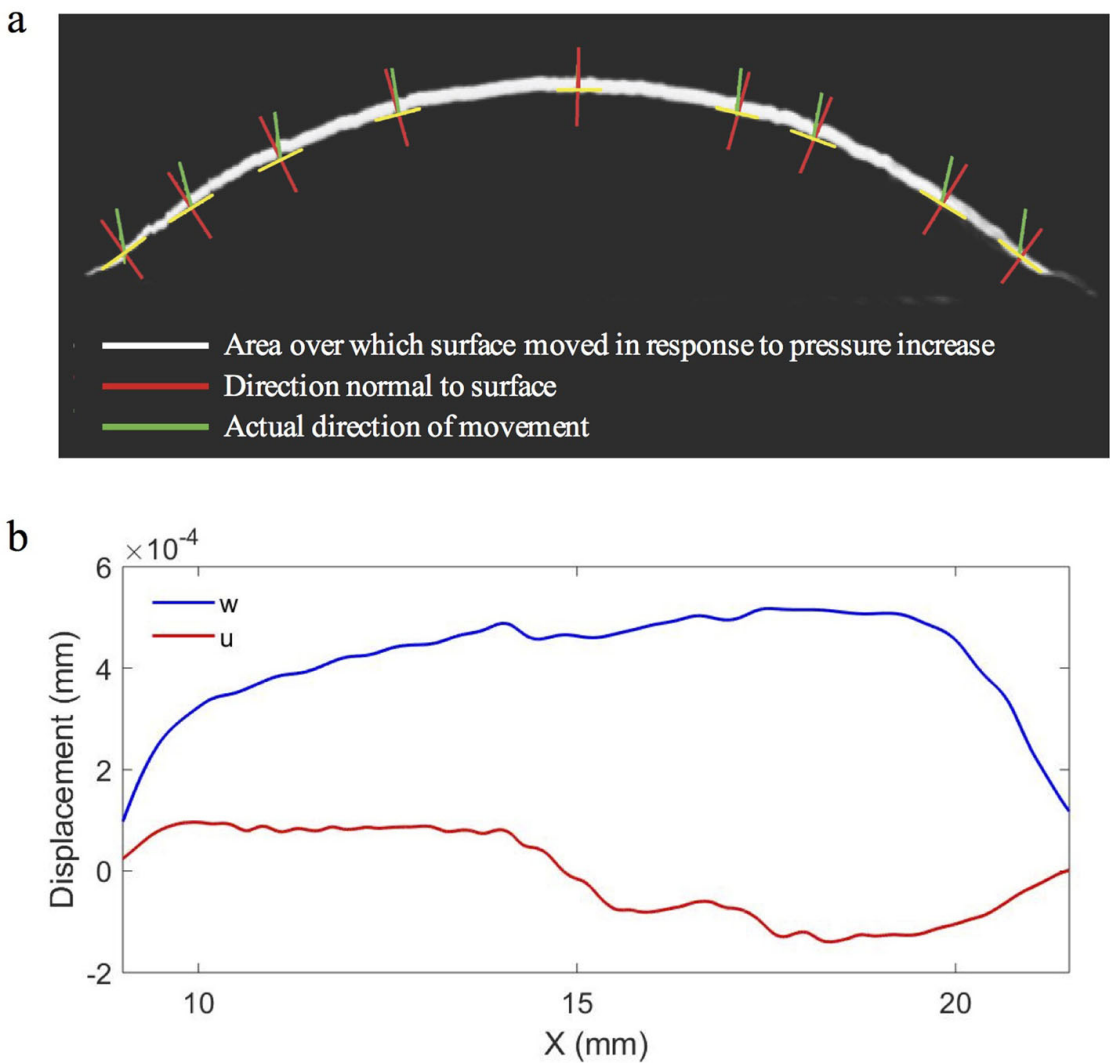
Subtractive video recording of the central cross-section of a cornea deforming in response to a pressure increase (**a**), comparison of measured out-of-plane and estimated horizontal in-plane displacement along central N-T cross section of a human cornea (**b**)

To examine the directional aspects of the response, the deformation of the central-cross-section of the corneas in response to a larger pressure change (< 20 mmHg) was recorded (Fig. 5). From this, it was identified that displacement tended to occur predominantly out-of-plane in comparison to that which would be predicted for an isotropic hemisphere where it would be expected to occur in a direction normal to the surface. The estimated in-plane component calculated using the angle of deformation and the measured out-of-plane component is shown in Fig. 5b. The in-plane contribution to overall displacement was relatively small, especially over the central 3 mm of the cornea and was approximately uniform either side of this over the central ~ 8 mm of the cornea. Hence, $\frac{\delta u}{\delta x}\approx 0$ over this region, and thus the measured differences in the out-of-plane displacement component may be considered predominately representative of changes in central curvature.

## Discussion

**Fig. 6:**
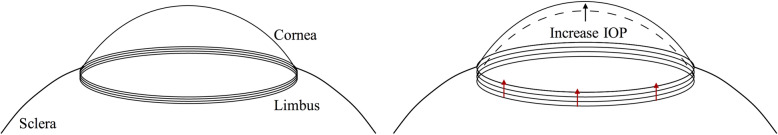
Hypothesised predominant mode of deformation in response to small pressure perturbations

The distribution of the observed displacement indicates that the biomechanics of the limbal region are fundamental to the response of the cornea to small pressure perturbations. Based on the data presented here, it is postulated that the limbus behaves akin to a dashpot, straining in the direction perpendicular to the circumferential orientation of collagen fibres in this region to absorb pressure increases (Fig. 6), facilitating the central optical zone to maintain a relatively constant curvature. Professor Keith Meek has previously put forward an identical hypothesis of limbal deformation (unpublished) to that shown in Fig. 6 as a result of his structural studies, but the present paper has been the first to provide empirical data on human cornea to support this mode of deformation. In addition, several previous studies have demonstrated similar evidence of a region of increased compliance approaching the limbal junction [17, 18, 30–32], whilst others have confirmed minimal changes occur to central corneal curvature in response to IOP changes [33, 34].

Structural aspects, including collagen distribution and orientation; and the distribution and concentration of elastin fibres, appear to support this mode of deformation. Both human and porcine corneas have an annulus of circumferentially aligned collagen at the limbus [35], an arrangement which would be expected to provide high circumferential strength to support the propensity towards out-of-plane motion (Fig. 5a) observed at the edge of the cornea. The increased thickness and collagen density in this region would also indicate that it is adept for dealing with stress, as the increased thickness of the tissue acts to minimise strain concentrations. However, strains were not quantified directly in this study due to an absence of through thickness information. An initial concern was that the fixed boundary constraints imposed on the sclera could have affected the motion of the cornea in regions within close proximity to the chamber. However, the experiments with a homogenous membrane did not indicate an obvious effect (Fig. 2) and results from a study by Smolek [31] on human eye globes using holography, showed similar deformation patterns for corneas that remained as a part of the eye globe.

Collagen crimp is a further feature of the cornea that has recently been postulated to play a key role in its mechanical behaviour [36], with a degree of fibrillar elongation via straightening of collagen crimp suggested to act complementary to the action of elastic fibres in the accommodation of small pressure perturbations [36]. Crimp parameters have been shown to vary significantly across the cornea [37, 38], suggesting a non-uniform response to loading, but studies have yet to examine regional modifications to collagen crimp under loading.

With respect to elastin fibres, studies have shown that they are most concentrated and thickest in the posterior limbus and peripheral cornea [39–41]. Here, they are present as elastin sheets, most concentrated in a layer above Descemet’s membrane, likely originating from the posterior limbus and adjacent sclera. From these sheets narrower fibres then extend towards the central cornea. The majority of fibres are reported to run radially or obliquely towards the limbus whilst others have been reported to run parallel to the circumferentially aligned collagen at the limbus [40]. Both the presence of elastin fibres and the elastin content has been confirmed to decrease towards the central regions of the cornea [41], again indicating that both the limbus and the peripheral cornea likely play a key role in the compensation of small pressure fluctuations.

Two additional aspects of the response that were highlighted from the displacement maps obtained on human and porcine corneas were differences in the behaviour of the superior-inferior (S-I) axis and nasal-temporal (N-T) axis, and a lack of symmetry in the response with respect to each axis. For human corneas, the magnitude of displacement across the N-T axis changed very little inside the limbus, whereas for the S-I axis, the magnitude of displacement very gradually increased towards the centre, suggesting that this axis was slightly less resistant to changes in curvature. Structural studies have identified differences between these two axes. Visually, it can be seen that there is an extension of limbal tissue onto the cornea at the top and bottom of the S-I axis, making it appear shorter than the N-T [42]. Differences have also been identified in the transitioning of collagen at the limbus in human corneas [43]. The N-T axis, having a 1.5 mm transition zone where the collagen running parallel to this axis, bifurcates to gradually become circumferentially aligned, whereas in the S-I axis, the collagen runs parallel to this axis right up until the limbus. Porcine corneas, have been reported in one study to lack the orthogonal arrangement of collagen across the central cornea [35], favouring a circumferential arrangement across the tissue. This could partially account for some of the differences seen in the response between human and porcine corneas. Based on the previous discussion, it would be of interest to examine differences, if any, in the elastic fibre network at the limbal junction with respect to these axes.

In terms of axial symmetry, it was evident, in most of the human corneas, that greater deformation tended to occur towards one side of the N-T axis, the specific side remains ambiguous as the position of the specific poles with respect to each axis could not be confirmed. Similarly, previous structural studies have shown that left and right eyes are structurally distinct [44], indicating that the biomechanics are unlikely to be axially symmetrical.

The specific features of the corneal response to small pressure changes that have been described here are highly important and require further investigation across a larger number of human samples. The information that has been demonstrated from this study and could be obtained from future studies using these imaging techniques, has the potential to enhance the accuracy and validity of computational models of corneal biomechanics aimed at predicting the outcomes of surgical procedures and clinical therapies such as CXL.

The non-destructive nature and high sensitivity of the measurement technique used in this study makes it a highly effective method for ex vivo examination of the regional changes to corneal biomechanics that occur due to invasive procedures such as refractive surgery, and due to treatments aimed at introducing biomechanical changes such as CXL. The latter could benefit greatly from spatial quantification of changes to biomechanics as this could enable the development of optimised treatment algorithms to deliver accurate and predictable refractive modifications.

A limitation of the method used is that it only measures surface displacements and therefore does not provide a full assessment of biomechanics. Other techniques have demonstrated compressive strains through the thickness of the cornea in response to physiological loading [15]. Hence, to gain a complete understanding of corneal biomechanics, it may be most effective to use this technique in combination with others, such as OCE or HFU, capable of measuring through-thickness displacements, while maximising the advantages of fast whole-field image acquisition times provided by interferometry.

A further limitation of this study, common with the majority of ex vivo studies, is the challenge in replicating and maintaining physiological levels of hydration in the tissue. Generally, human corneal samples cannot be obtained fresh and storage results in corneal swelling. Since hydration contributes to tissue biomechanics, swelling could lead to changes to the response of the tissue. However, a study had demonstrated only slight differences in deformation patterns with IOP despite significant differences in hydration state [45], and it would be expected that even if changes in the magnitude of deformation changed, the overall mechanism of deformation, with high strain concentrations at the limbus, would remain the same.

Although this study has been ex vivo in nature, it ultimately is necessary for information of this kind, regarding spatial variation in biomechanics, to be measured in vivo. This would facilitate the early detection and optimised treatment of diseases, such as keratoconus, prior to significant changes in topography; and enable individuals that may be at risk of post-LASIK ectasia to be identified, preventing them from undertaking potentially damaging elective treatments. Further, if an individual’s corneal biomechanics could be topographically mapped, it would offer the opportunity for delivery of optimised and customised procedures such as laser refractive surgery for astigmatism, and CXL together with adjustments post-cataract astigmatism. Significant efforts are going into the development of a clinical device based on similar principles.

## Conclusions

The stiffness of the cornea is regionally variable. The limbal and pre-limbal regions of the cornea appear to play a key role in the absorption of small pressure fluctuations, facilitating the central cornea to maintain a relatively constant curvature. These findings could have important implications for corneal surgery and for the delivery of targetted-CXL, hence further investigations are required on a larger number of human samples.

DSPI has been demonstrated here as an effective technique to examine corneal surface deformation in response to physiological scale pressure variations, and can be used alongside other methods to map corneal biomechanics.

## Supplementary information

**Additional file 1: Supplementary Figure 1. ** Diagrammatic summary of the working principles of displacement speckle pattern interferometry (DSPI).

**Additional file 2: Supplementary Figure 2.** Surface plots showing out-of-plane displacement of human corneas in response to a pressure increase from 16.50 mmHg to 16.75 mmHg. Positions marked with **X** relate to locations at which limbal and central displacements were compared in Table 1.

**Additional file 3: Supplementary Figure 3.** Typical response of a porcine cornea to a pressure increase from 16.50 mmHg to 17.00 mmHg. Full surface map of out-of-plane displacement (a), out-of-plane displacement along section A-A (b), out-of-plane displacement along section B-B (c).

**Additional file 4: Supplementary Figure 4.** Measurement region damaged due to insertion of surgical thread; photograph of cornea whilst suspended in transplant solution (a); map of out-of-plane surface displacement in response to pressure increase from 16.5 mmHg to 17.0 mmHg (b), area of damage clearly evident as region of increased displacement (dark red region).

## Data Availability

All relevant data generated or analysed during this study are included in this submitted article and its supplementary files. Full imaging datasets are available from the corresponding author on reasonable request.
