# Supplementary figures and images for: An interferometric ex vivo study of corneal biomechanics under physiologically representative loading, highlighting the role of the limbus in pressure compensation
Source: Eye Vis (Lond). 2020 Aug 13;7:43. doi: 10.1186/s40662-020-00207-1 (PMC7433364; doi:10.1186/s40662-020-00207-1)

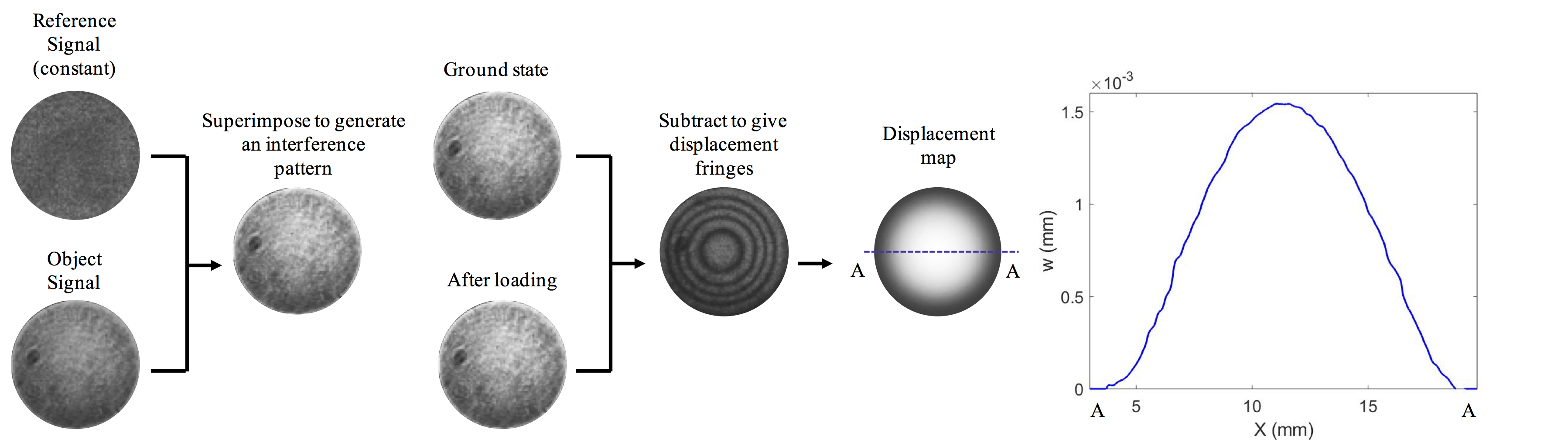

Supplement: Supplementary file 1 — Additional file 1: Supplementary Figure 1. Diagrammatic summary of the working principles of displacement speckle pattern interferometry (DSPI). [file 40662_2020_207_MOESM1_ESM.jpg]

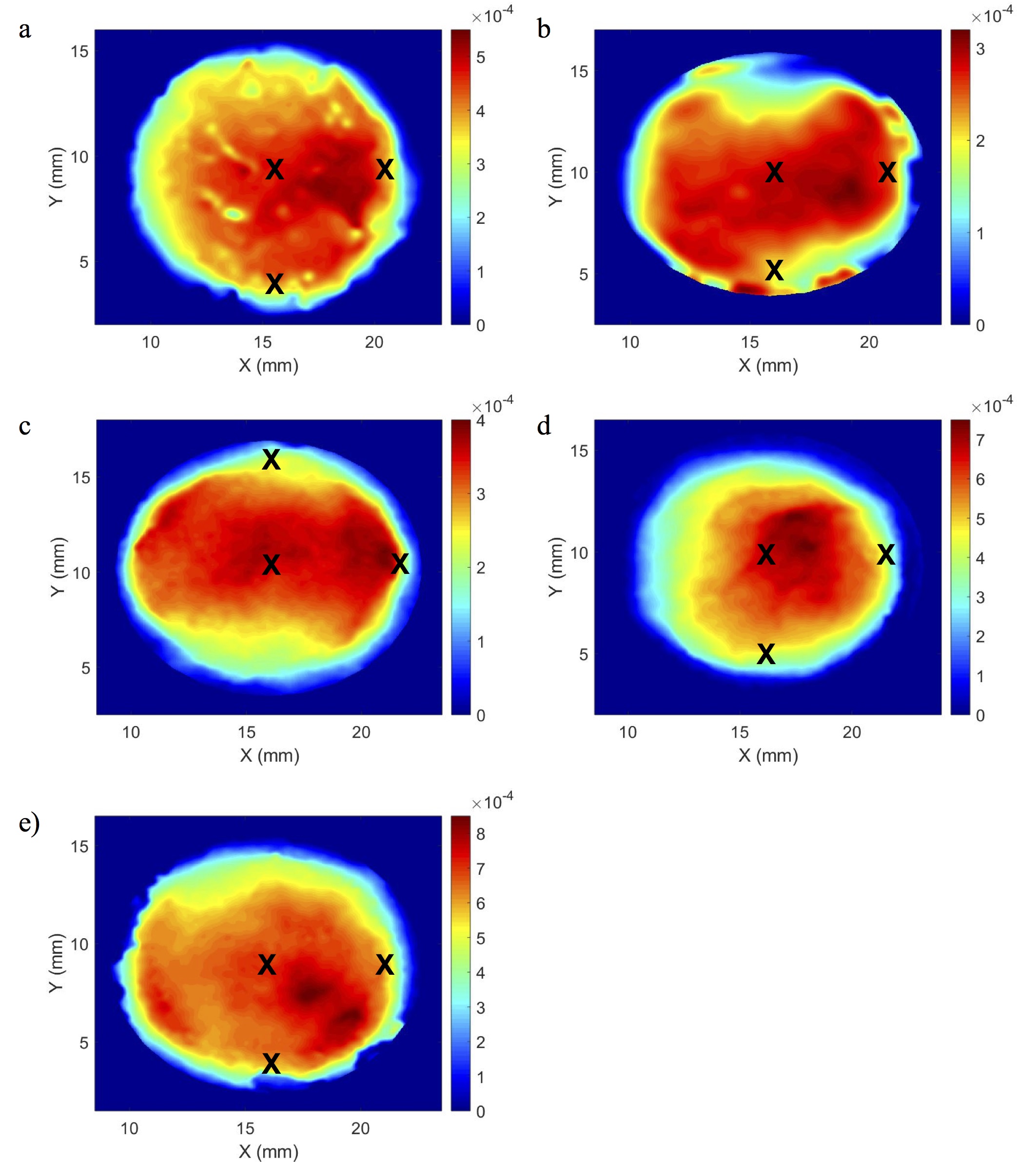

Supplement: Supplementary file 2 — Additional file 2: Supplementary Figure 2. Surface plots showing out-of-plane displacement of human corneas in response to a pressure increase from 16.50 mmHg to 16.75 mmHg. Positions marked with X relate to locations at which limbal and central displacements were compared in Table 1. [file 40662_2020_207_MOESM2_ESM.jpg]

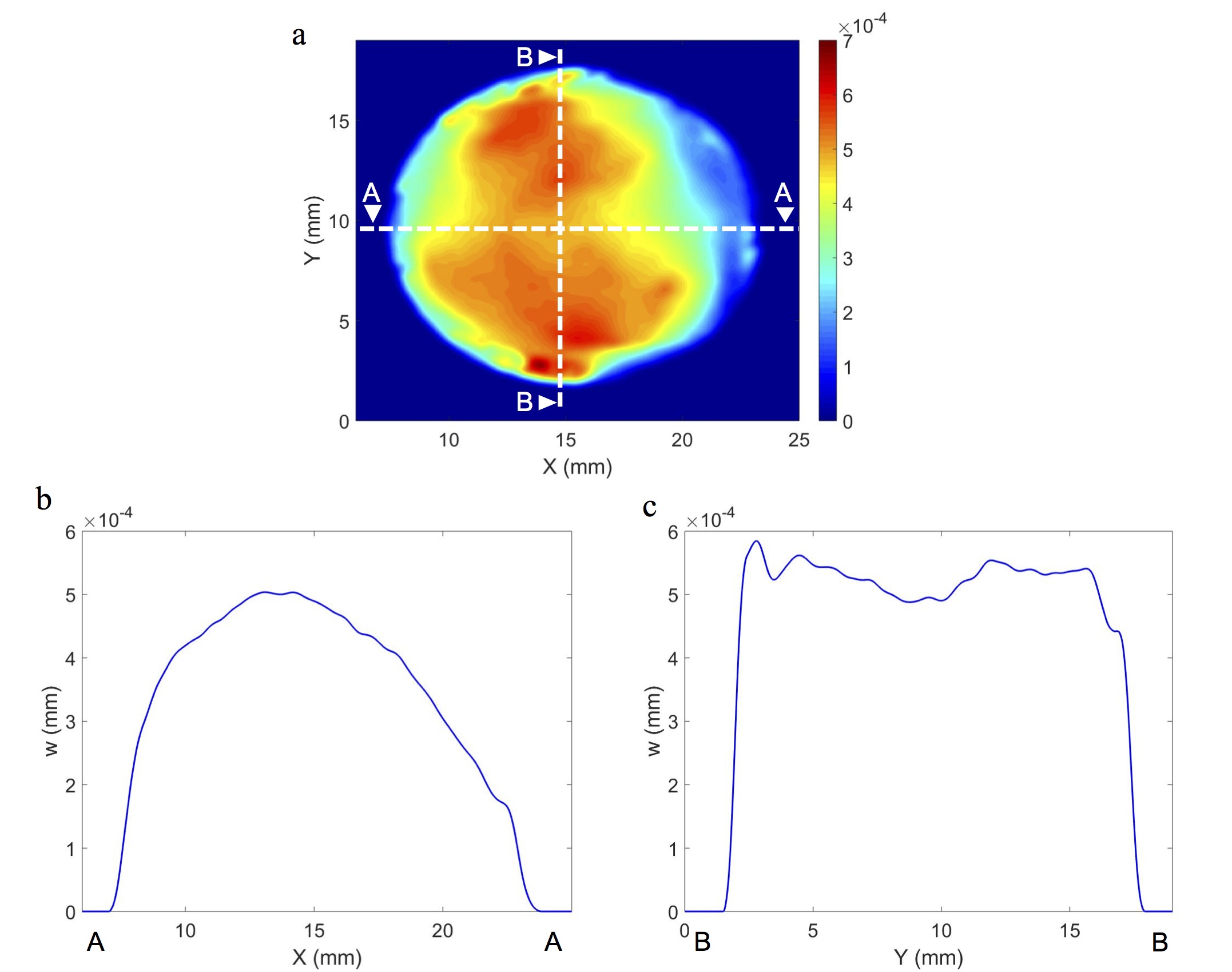

Supplement: Supplementary file 3 — Additional file 3: Supplementary Figure 3. Typical response of a porcine cornea to a pressure increase from 16.50 mmHg to 17.00 mmHg. Full surface map of out-of-plane displacement (a), out-of-plane displacement along section A-A (b), out-of-plane displacement along section B-B (c). [file 40662_2020_207_MOESM3_ESM.jpg]

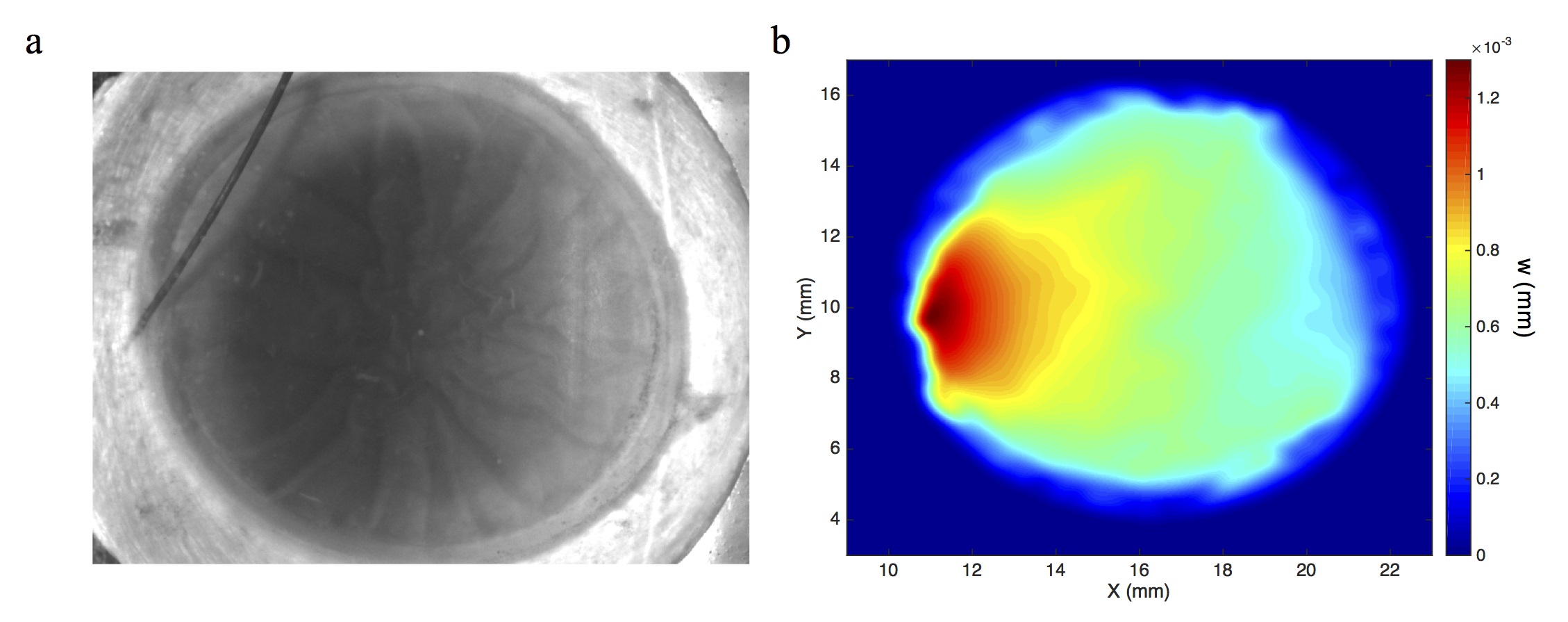

Supplement: Supplementary file 4 — Additional file 4: Supplementary Figure 4. Measurement region damaged due to insertion of surgical thread; photograph of cornea whilst suspended in transplant solution (a); map of out-of-plane surface displacement in response to pressure increase from 16.5 mmHg to 17.0 mmHg (b), area of damage clearly evident as region of increased displacement (dark red region). [file 40662_2020_207_MOESM4_ESM.jpg]
